# Knowledge gaps in implementing global mental health activities

**DOI:** 10.1017/gmh.2017.20

**Published:** 2017-11-27

**Authors:** S. Saxena, G. Belkin

**Affiliations:** 1 Director, Department of Mental Health and Substance Abuse, World Health Organization, Geneva, Switzerland; 2 Editor in Chief, Global Mental Health, New York City, NY

Global mental health has rapidly attracted increasing attention by health policy makers and researchers during the last 10 years. The groundwork for this shift was laid in data being made available on the prevalence (Demyttenaere *et al*. 2004) and burden (Lopez *et al*. 2006) of mental disorders and also on the resources allocated to mental health within countries (WHO, 2001, 2005, 2011, 2015). Publication of a series of review papers on specific areas within global mental health (Lancet Series on Global Mental Health, 2007, 2011) and on Grand Challenges in Global Mental Health (Collins *et al*. 2011) focused attention on what was known and also on what were the gaps in knowledge. On the side of policy makers, mental health has been discussed at ministerial level in the Commonwealth, the Asia-Pacific Economic Commission, and in the World Health Assembly consisting of 194 ministers of health. The latter discussion led to the adoption of Comprehensive Mental Health Action Plan 2013–2020 (WHO, 2013), first time in the history of World Health Organization.

The Mental Health Action Plan of WHO captures this global engagement, and is clearly focused on action. It has four objectives: to improve leadership and governance, health and social care, promotion and prevention, and information and research. It also identifies key cross-cutting principles encompassing universal health coverage, human rights, evidence based practice, life-course approach, multi-sectoral approach and empowerment of persons with mental disorders and psychosocial disabilities. The Action Plan is based on the vision of ‘a world in which mental health is valued, promoted and protected, mental disorders are prevented and persons affected by these disorders are able to exercise the full range of human rights and to access high-quality, culturally-appropriate health and social care in a timely way to promote recovery, all in order to attain the highest possible level of health and participate fully in society and at work free from stigmatization and discrimination’ The Plan calls for specific actions to be taken by member states, WHO secretariat and international and national partners. As the action plan is getting implemented, world leaders have also made an even larger commitment to mental health by including it in the United Nations Sustainable Development Goals 2015–2030. This places mental health within the global development agenda.

Do we have all the knowledge that we need to implement the Plan or the mental health and wellbeing component of UN Sustainable Development Goals? The clear answer is no. Existing knowledge has been critical in setting the high-level agendas for action, but there remain knowledge gaps that need to be filled. Nonetheless, having these global goals and shared aims is an important start to advance and align growing research activity in mental health services design and implementation, especially in low- and middle-income countries (LMICs). Efforts by researchers and innovators supported by funders like the Grand Challenges Canada (GCC website), National Institute of Mental Health of USA (NIMH Hubs website), Department of International Development of UK (PRIME), European Commission (Emerald) are extremely relevant towards this objective. In addition, a number of researchers are working towards generating and aggregating critical evidence that will help in achieving the objectives of global commitments.

This issue of Global Mental Health is intended to shed light on some of the evidence and experience that are relevant to implementing each of the five key objections of the Action Plan. Those objectives are to: (1) update mental health laws in line with international human rights instruments; (2) improve service coverage for serious mental illness by 20%; (3) have at least two functioning multi-sectoral prevention/promotion programmes; (4) reduce national suicide rate by 10%; and (5) establish minimal biennial national core dataset collection and reporting. For each of these objectives we present overview/review papers that summarize the research literature relevant to meeting that objective, and original research papers that look at current areas of innovation and attention. This issue we hope therefore contributes towards filling the knowledge gaps for implementers working to reach the Action Plan objectives, as well as pointing out the needs and opportunities ahead in creating the learning to meet these goals.

Taking up each of these objectives in turn, first Marianne Schulze provides an overview of human rights principles for developing policies and laws on mental health, highlighting the need for all policies and laws to conform to international human rights agreements like the UN Convention on the Rights of Persons with Disabilities. Szmukler & Bach (2015) flesh that out further with a particular focus on the UN Convention on the Rights of Persons with Disabilities and its place within other international human rights instruments with respect to mental illness.

The idea of ‘task sharing’ or task-shifting describes the idea of optimizing the skillsets of non-specialized workers to provide much of the steps in mental health care and promotion, such as through community health workers. It is increasingly turned to as a model for achieving the expanded service coverage goal of the Action Plan, and closing treatment gaps more broadly. The paper by Javadi *et al.* (2017) provides a critical review of the current literature on task-sharing for mental health. Kohrt *et al*. (2015) then further focus on more recent work that evaluates the clinical competence of primary care providers to deliver mental health care within the paradigm of task sharing. While task sharing remains a key strategy to scale up care, these papers identify challenges in its execution, especially the opportunity areas for further research on those challenges, such as the evaluation and ongoing support of competencies and outcomes, and therefore of taking this approach to scale to meet Action Plan objectives.

In terms of prevention and promotion, Tol (2015) provides an overview of work in this area to date, while Glenshaw *et al*. (2016) describe several levels of public health response across two countries designed to diminish and prevent the population burden, in this case specifically focusing on alcohol use. The paper by Fleischmann *et al*. (2016) summarizes evidence for effective interventions for suicide prevention. Suicides are responsible for more than 800 000 deaths globally. WHO's Action Plan has the target of 10% reduction in suicide rate till 2020, making this evidence extremely critical for countries.

Finally, timely and actionable information is a critical element in better planning. Lora & Sharan (2015) provide an overview of the progress made in countries with support from WHO in national data gathering and surveillance, and also the need for greater efforts in this area. That review is paired with a look at new work in the field of mobile phone-based support for clinical care discussed by Maulik *et al*. (2015) in a study being conducted in rural communities. If found effective, this kind of strategy can contribute to scaled gathering as well as play a multiplier role in providing care to communities that often have no care at all. Arjadi *et al*. (2015) extend those possibilities for linking the potential of online and mobile extensions of care and support to data gathering by providing a review of the prospects of online interventions in LMICs.

These papers, though not the last word in identifying knowledge gaps on the path towards achieving the goals of the WHO Action Plan, do illustrate how much we do know to build on moving forward, as well as where to sharpen future efforts in generating, aggregating and disseminating information and evidence for more effective implementation of global mental health activities.
